# Supplementary figures and images for: Mitochondrial apolipoprotein MIC26 is a metabolic rheostat regulating central cellular fuel pathways
Source: Life Sci Alliance. 2024 Oct 11;7(12):e202403038. doi: 10.26508/lsa.202403038 (PMC11472510; doi:10.26508/lsa.202403038)

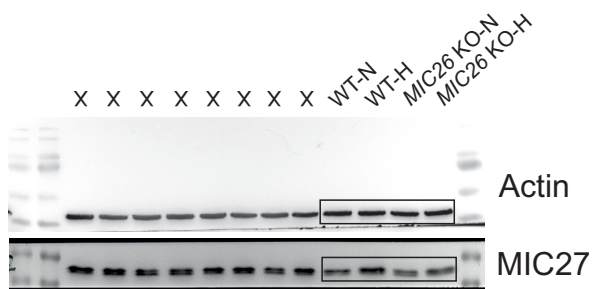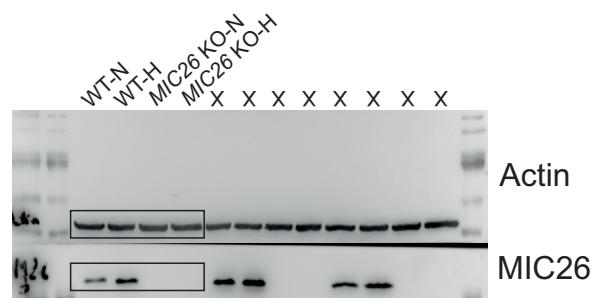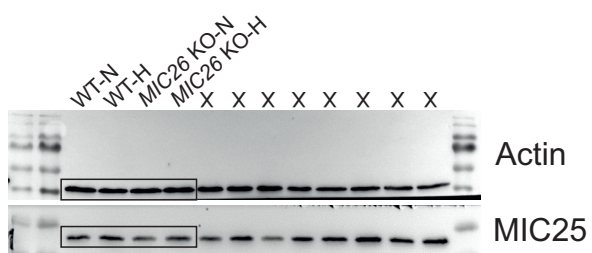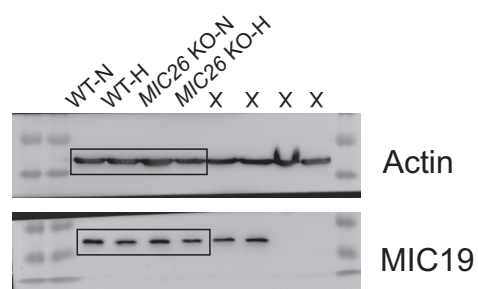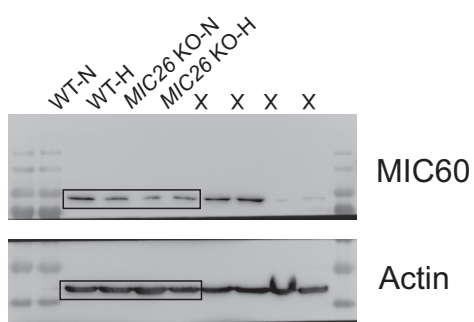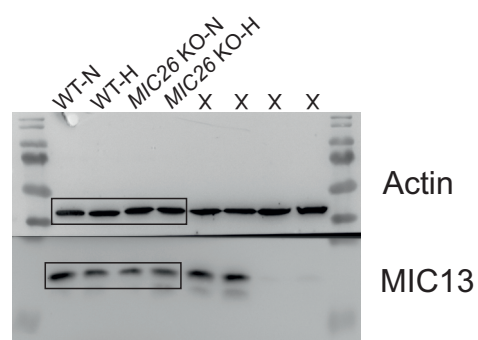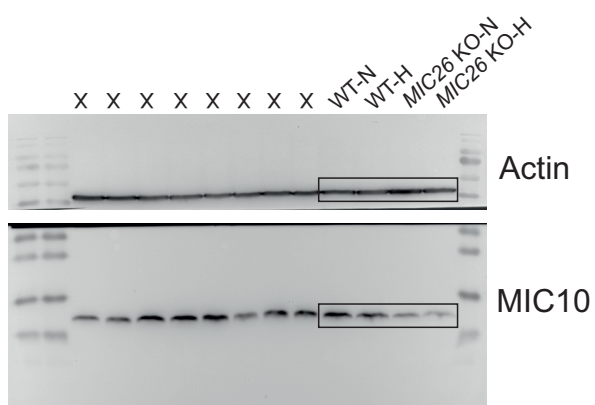

Supplement: Supplementary file 1 [file LSA-2024-03038_SdataF1.pdf]

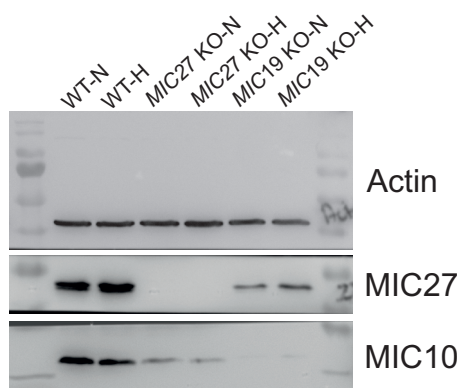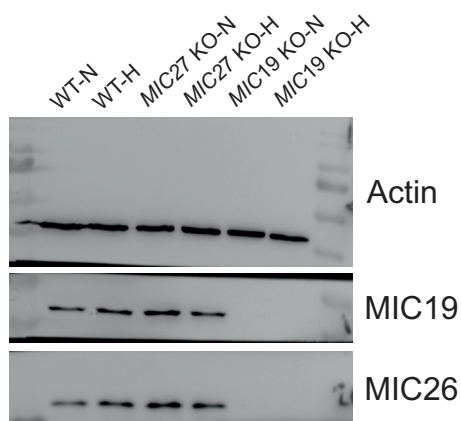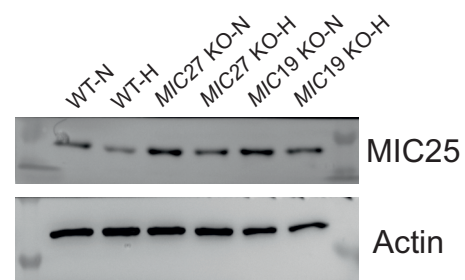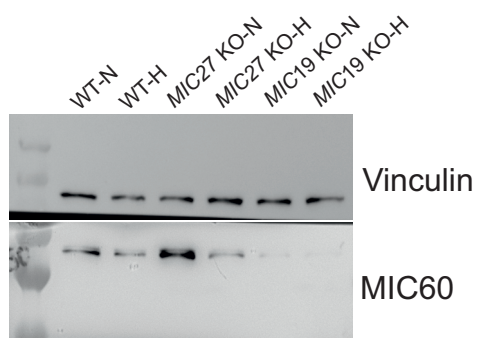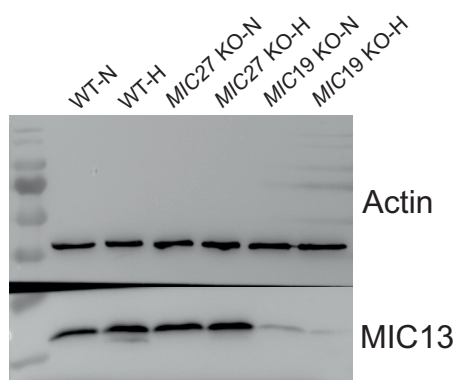

Supplement: Supplementary file 2 [file LSA-2024-03038_SdataFS4.pdf]

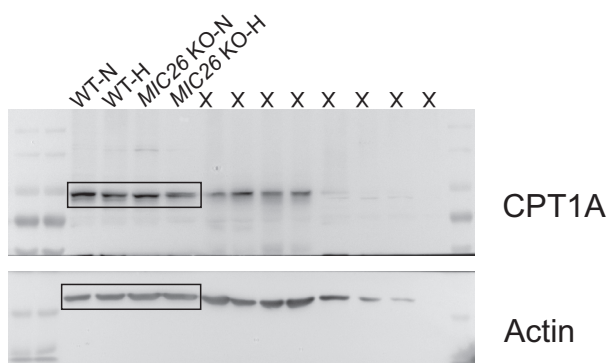

Supplement: Supplementary file 3 [file LSA-2024-03038_SdataF4.pdf]

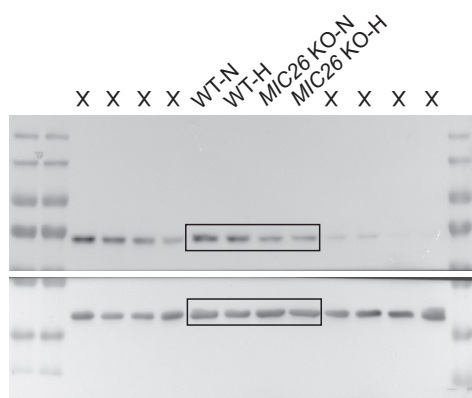

SLC25A12

Actin

Supplement: Supplementary file 4 [file LSA-2024-03038_SdataF6.pdf]

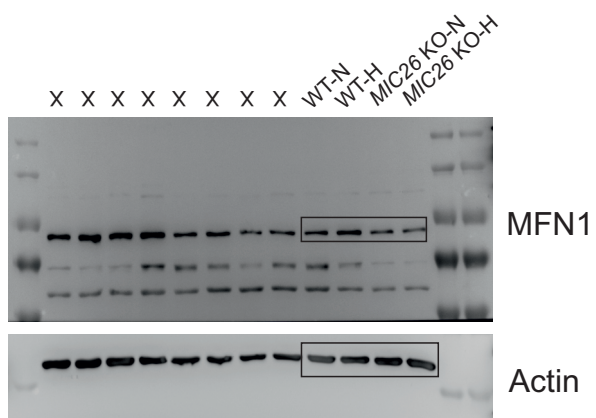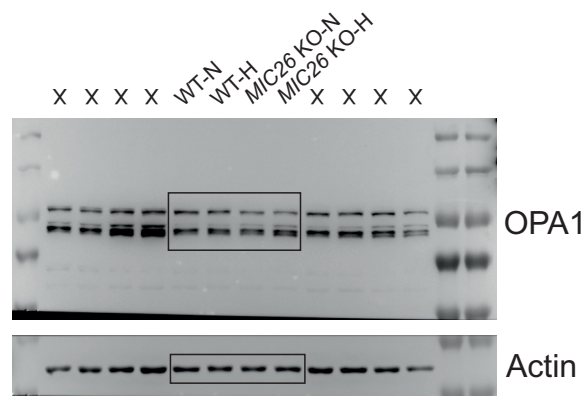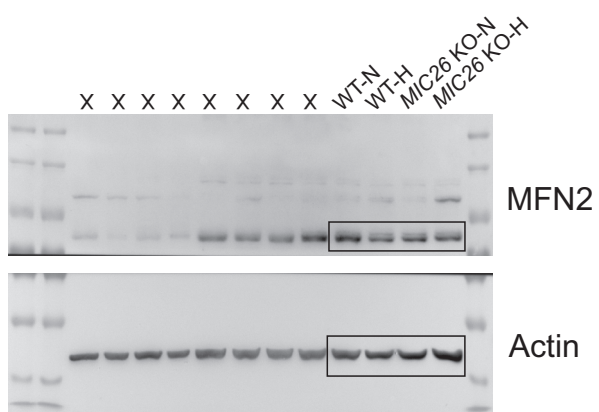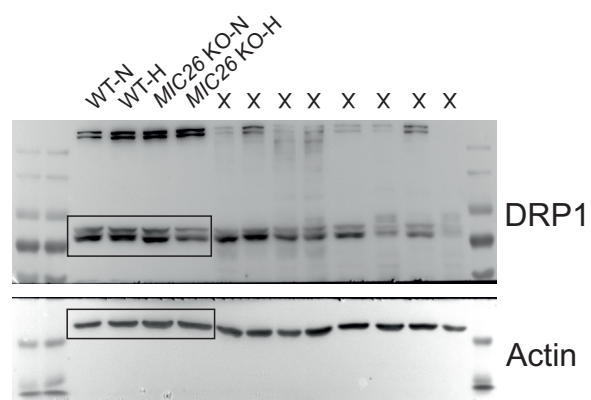

Supplement: Supplementary file 5 [file LSA-2024-03038_SdataFS11.pdf]
